# Safety of Nicotine Replacement Therapy during Pregnancy: A Narrative Review

**DOI:** 10.3390/ijerph20010250

**Published:** 2022-12-23

**Authors:** María Morales-Suárez-Varela, Beatriz Marcos Puig, Linda Kaerlev, Isabel Peraita-Costa, Alfredo Perales-Marín

**Affiliations:** 1 Area of Preventive Medicine and Public Health, Department of Preventive Medicine and Public Health, Food Sciences, Toxicology and Forensic Medicine, University of Valencia, Av. Vicente Andrés Estellés s/n, 46100 Burjassot, Spain; 2 CIBER in Epidemiology and Public Health (CIBERESP), Av. Monforte de Lemos, 3–5 Pabellón 11, Planta 0, 28029 Madrid, Spain; 3 Department of Obstetrics, La Fe University Hospital, Av. Fernando Abril Martorell 106, 46026 Valencia, Spain; 4 Research Unit of Clinical Epidemiology, Institute of Clinical Research, University of Southern Denmark, Odense, J.B. Winsløws Vej 19, 3, 5000 Odense, Denmark; 5 Research Unit of Clinical Epidemiology, Center for Clinical Epidemiology, Odense University Hospital, Kløvervænget 30, Entrance 216 Ground Floor East, 5000 Odense, Denmark

**Keywords:** smoking/harm reduction, cessation, nicotine or derivatives, therapeutic use, prenatal exposure

## Abstract

Background: Smoking during pregnancy is a public health problem worldwide and the leading preventable cause of fetal morbidity and mortality and obstetric disease. Although the risk of tobacco-related harm can be substantially reduced if mothers stop smoking in the first trimester, the proportion of women who do so remains modest; therefore, the treatment of smoking in pregnant women will be the first therapeutic measure that health professionals should adopt when providing care to pregnant women. The recommendation of nicotine replacement therapy during pregnancy remains controversial due to the potential effects on the health of the fetus. Purpose: The aim of this review was to provide an overview of human studies about the use of nicotine replacement therapy during pregnancy, evaluating the efficacy and safety of the different formulations. Methods: The electronic databases PubMed and EMBASE were searched from May 2012 to May 2022. A total of 95 articles were identified through database searching using a combination of keywords. Out of 79 screened articles and after the removal of duplicates, 28 full-text articles were assessed for eligibility and 12 articles were finally included for review. Results: Although demonstrated to be effective in adult smokers, evidence in support of NRT in pregnant women is limited. The results of the apparent safety of the use of NRT during pregnancy contradict the FDA classification of the different NRT formulations. Faster-acting formulations seem to be the safest and even most beneficial forms for the offspring. Conclusions: NRT is not completely harmless for the fetus or for the mother; however, if an adequate assessment of the risk-benefit binomial is made, its use during pregnancy to aid in quitting smoking does seem appropriate. It is necessary to establish individual recommendations on the formulation and dose to be used during pregnancy based on individual nicotinic needs.

## 1. Introduction

Smoking during pregnancy is associated with different serious adverse obstetric and neonatal outcomes [1,2]. These health risks to the developing fetus will create problems that will persist long after delivery [3]. It is estimated that the prevalence of maternal smoking ranges between 10.9% and 38.4% in Europe [4]. Available data suggest that between 15 and 20% of all pregnant women will continue to smoke during pregnancy, and while the prevalence has decreased in high-income countries, it continues to increase in low- and middle-income countries [5]. Although the risk of tobacco-related harm can be substantially reduced if mothers quit smoking in the first trimester, the proportion of women who do so remains modest [6,7,8,9,10]. Smoking and difficulty with quitting smoking during pregnancy have been shown to be more common in women from low-income families, with low educational levels, with a high number of children, with a partner or household members who smoke, who consume alcohol and/or who suffer depression [10].

Many women are unable to quit smoking despite receiving behavioral interventions [3,10]. It is in these cases that the use of nicotine replacement therapy (NRT) during pregnancy is recommended [9,11]. The efficacy of NRT in the general population of smokers is well studied; however, there is limited evidence on the efficacy and safety of these medications when used during pregnancy [8]. NRT is the most studied pharmacotherapy used for smoking cessation among pregnant women [2]. Nicotine acts on cholinergic nicotinic-type acetylcholine receptors (nAChr), such as ⍺4 β2, located in the membranes of neurons in the ventral tegmental area of the midbrain, and it leads to increased dopamine release in the nucleus accumbens and is responsible for the feeling of reward. 

On the one hand, the use of NRT to avoid smoking during pregnancy reduces the number of toxins (more than 4000 compounds, including nicotine, CO, carcinogens and heavy metals present in tobacco), but on the other hand, NRT exposes the nicotine [9] so its use during pregnancy has been controversial due to possible pulmonary consequences based on animal studies [12] and cognitive deficits in newborns after prenatal exposure to nicotine have been shown in animals and appear to last into adulthood [13,14]. Moreover, as a slower-release form of nicotine, NRT is much easier to get off of than cigarettes. However, the risk of relapse to cigarette smoking after delivery is very high, and close follow-up is needed. NRT would commonly need to be continued (or perhaps initiated) in the post-partum period to minimize the risk of relapse.

It should be noted that the pharmacokinetic characteristics of nicotine in the fetus are different from those in the mother. Most of the nicotine that reaches the fetus returns to the maternal circulation, so the mother is responsible for its elimination. However, the fetus excretes a certain amount of nicotine into the amniotic fluid through urine. This will contribute to the amniotic fluid of pregnant smokers having high concentrations of nicotine and cotinine. This fact causes the fetus to be exposed to high concentrations of nicotine even after concentrations in maternal blood have decreased [15,16]. Animal studies have shown that nicotine exposure during pregnancy causes adverse perinatal effects, as well as unfavorable endocrine, reproductive, respiratory, cardiovascular, and neurological system outcomes in offspring [17,18,19]. The suitability of these studies for direct clinical application is still questionable, as animal models differ in the form of nicotine exposure (nicotine in drinking water, via subcutaneous mini-pumps, exposure to tobacco smoke, etc.), some of which are stressful to the animal. These differences introduce important potential confounding factors. In addition, the metabolism of nicotine is different, as well as the development of the brain between humans and animals. It is for these reasons that animal studies will not be included in this review [20].

Despite several systematic reviews and recommendations on the benefit-risk ratio of NRT use during pregnancy, current knowledge is insufficient about the different types of NRT and the efficacy and safety of each of them for smoking cessation in pregnancy [3,9,21,22,23,24,25]. The aim of this study was to provide a review of published studies about efficacy and safety associated with different types of NRT administered during pregnancy.

## 2. Methods

### 2.1. Search Strategy

This review was performed according to PRISMA guidelines [26,27]. The electronic databases PUBMED and EMBASE were searched from 2012 to May 2022. The following keywords were used “nicotine replacement therapy” AND “pregnancy” AND “human” AND “newborn”.

### 2.2. Eligibility Criteria and Main Outcome Measures

No restrictions on language and publication status were applied. Abstracts were included if there was sufficient information to assess the study’s quality. Original studies that explored NRT during pregnancy in humans and review articles that aimed to determine the efficacy and safety of pharmacotherapy for smoking cessation when used during pregnancy were eligible for inclusion. The exclusion criteria were studies that did not mention pharmacotherapy or without mention of metabolism in pregnant women, those that did not study the use of different formulations of NRT among smokers and non-human research.

### 2.3. Data Extraction

Two authors conducted data extraction independently and reached a consensus. The first selection was made based on title and abstract. Eligible publication full texts were assessed. Variables of interest extracted were authors’ names, the year of publication, the type of study, the sample size, the period of inclusion, the country of the study, and the article’s main outcomes.

The different selected studies that met the inclusion criteria were classified according to their pharmaceutical form: chewing gum; inhalers; patches; combination of different NRT formulations. The information obtained for each of the different NRT formulations was assessed: efficacy and safety.

## 3. Results

A total of 95 articles were identified through database searching using a combination of keywords. Out of 79 screened articles and after duplicates were removed, 28 articles were assessed for eligibility. Finally, nine original articles were found and included in this review, along with three review articles. In all of the studies, the degree of smoking of the mothers is established either through questionnaires or through the determination of biological markers (cotinine and CO). In some of them, the degree of dependency (FTND and HSI) is also determined.

### 3.1. Single-Form NRT

In relation to NRT gum or lozenge, specifically on the use of nicotine gum, there is a study by Oncken et al., 2008 [28], which aimed to estimate the safety and efficacy of treatment with 2 mg nicotine gum to stop smoking during pregnancy. One hundred ninety-four pregnant women smokers who were in the 17th week of gestation received individualized behavioral counseling (two sessions, lasting 35 min) and were randomly assigned a six-week treatment of nicotine gum or placebo, followed by a reduction period of another six weeks participated. The results determined that biochemically validated smoking cessation rates were non-significantly higher with NRT than with placebo (after six weeks of treatment: 13% vs. 9.6%, *p*-value (*p*) = 0.45; at 32–34 weeks of gestation: 18% compared to 14.9%, *p* = 0.56). NRT, compared to the placebo group, showed significantly greater reductions in cigarettes smoked per day. Birth outcomes showed that there were clinically important and statistically significant differences in favor of NRT compared to placebo in birth weight (3.29 g [SD = 566] and 2.95 g [SD = 653], respectively; *p* < 0.001) and gestational age (38.9 weeks [SD = 1.7] and 38.0 weeks [SD = 3.3], respectively; *p* = 0.014). Nine times higher incidence of low-birth-weight infants and twice the incidence of preterm birth in the placebo group compared to the NRT group. In summary, nicotine gum therapy was associated with a lower risk of preterm birth and higher neonatal birth weight.

Concerning oral nicotine inhaler, it is a form of NRT that provides some of the sensory and ritualized components of smoking, such as handling and inhalation, which may be particularly important for women smokers and thus increase adherence to treatment in women. Absorption is through oral mucosae with this device, with an onset of action similar to NRT gum/lozenge. The study by Oncken et al., 2019 [29] is the only trial that examines the efficacy and safety of oral nicotine inhalers for smoking cessation during pregnancy. One hundred thirty-seven pregnant women (17 to 20 weeks) who smoked more than five cigarettes a day were randomly assigned to six weeks of treatment with a nicotine inhaler or a placebo, followed by a follow-up period of another six weeks. The nicotine and placebo groups were comparable for most demographic, smoking, medical, and obstetrical history variables; however, there were baseline differences in motivation to quit. The results were not significantly different by treatment assignment; at six weeks, 3 of 70 participants in the nicotine group (4%) compared to 2 of 67 in the placebo group (3%) had remained abstinent (*p* > 0.99). Even the dropout rate was higher at 32 weeks’ gestation for the placebo group (12 of 67, or 18%) than for the nicotine group (7 of 70, or 10%) (*p* = 0.220). A difference in initial motivation to quit smoking could explain this difference in quit rates at 32 weeks gestation between the two groups. However, it should be noted that although abstinence was not achieved in most of the pregnant women who participated in the study, a reduction in the number of cigarettes smoked per day was observed from the beginning of the trial to the end in the group randomized with nicotine. With regard to safety in nicotine oral inhalers, more women in the placebo group had a preterm birth compared to the nicotine group (15% (10 of 67) vs. 4% (3 of 67), respectively, *p* = 0.030) or a low-birth-weight baby (15% (10 of 67) vs. 6% (4 of 67), respectively, *p* = 0.035). On the other hand, a group of participants who had been randomized to NRT reported moderate adverse reactions, all associated with the use of oral nicotine inhalers during treatment, including throat irritation, bad taste, and increased mucus in the upper respiratory airways, which are well known. Throat irritation likely arises from attempts to inhale like a cigarette rather than holding the dose in the mouth to absorb through oral mucosae. These findings suggest that oral nicotine inhaler has a favorable safety profile.

Nicotine patches are the most studied presentation of NRT in recent times, Coleman et al. 2012 [30] in a randomized multicenter study in which 1050 pregnant women from 12 to 24 weeks and who were smokers of at least five cigarettes per day participated. Participants were randomly assigned eight weeks of treatment with active nicotine patches or placebo patches. The results determined that there was no significant abstinence rate from the date pregnant women committed to quitting until delivery between the NRT and placebo groups (9.4% and 7.6%, respectively; OR 1.26; CI 95% of 0.82–1.96), although the rate of smoking cessation among study women in the first month in the NRT group was higher than in the control group (21.3% vs. 11.7%: OR 2.05, CI 95 of 1.46–2.88). Of note, compliance was low (only 7.2% of women assigned to NRT and 2.8% assigned to placebo used patches for more than one month). Regarding the safety of NRT, birth outcomes were similar between both groups. Of note, there were significantly more cesarean deliveries in the NRT group than in the placebo group (20.7% vs. 15.3%). The interpretation of the absence of apparent harm from NRT as an indication of safety is warranted by the trial with caution, given the low rates of adherence to therapy and the fact that a larger sample would be needed to assess the effect of NRT regarding adverse effects comprehensively.

In the trial conducted by Berlin et al., 2014, [31] 402 pregnant women participated (a smaller sample than in the Coleman trial) who were randomized: 203 received nicotine patches and 199 placeboes, and both groups received behavioral support. Identical nicotine and placebo patches were administered from the day of smoking cessation until the time of delivery. Nicotine patch doses were adjusted for salivary cotinine levels during smoking to obtain a 100% substitution rate. The two main differences with respect to the trial by Coleman et al., 2012 [30], were that, in the previous one, doses of up to 15 mg of nicotine per day were evaluated, and the trial had an exposure duration of no more than eight weeks. The results showed: complete abstinence and point prevalence of abstinence, time to drop or relapse. Complete abstinence was achieved in 11 women in the nicotine patch group (5.5%) and 10 women in the placebo group (5.1%) (OR 1.08, 95% CI 0.45–2.60). The median time to the first cigarette smoked after the target quit day was 15 days in both groups, and point prevalence abstinence showed no statistically significant difference between nicotine patches (8% to 12.5%) and placebo (8% to 9.5%). Compared to the other studies, the compliance rate was much higher. This study showed that diastolic pressure (DP) was significantly increased in the nicotine patch group compared to the placebo patch group in late pregnancy, which could lead to unfavorable pregnancy outcomes. Regarding the rest of the adverse events, as in the trial by Coleman et al., 2012 [30], Berlin et al. [31] did not find significant differences in adverse events between the placebo group and the nicotine patch group and, in the same way as in the other study, their conclusions are not completely certain since it was considered that this would have required a group of women greater than the employed to carry out the study.

The trial by Coleman et al., 2012 [30], did not study the subsequent effects of NRT in the children of mothers treated with nicotine patches, but it is from this that Cooper et al., 2014 [32], evaluate whether the use of NRT in pregnancy could harm child development two years after delivery. To assess the safety variable, the participants received questionnaires at 6, 12 and 24 weeks after delivery. Collection and interpretation of the questionnaires showed that children born to women who used NRT to quit smoking during pregnancy were more likely to have a smooth development (in the NRT group, 323 (73%) of 445 babies had no deterioration compared with 290 (65%) of 443 infants in the placebo group (OR 1.40, 95% CI 1.05–1.86, *p* = 0.023). However, no difference in frequency was observed with respect to respiratory problems.

Five years later Iyen et al., 2019 [33] investigated whether the absence of developmental deficits in babies two years after birth, as concluded in the study by Cooper et al., 2014 [32], was associated with maternal smoking status (which was measured at different points in the trial). No association was found between maternal smoking status at different times of pregnancy and developmental impairment in infants at two years. Consequently, there was no evidence to support the hypothesis that the better infant development observed within infants born to women who were randomized to NRT in the trial by Coleman et al., 2012 [30] was the result of induced smoking cessation from the use of nicotine patches.

### 3.2. Combination NRT

The combined use of NRTs (generally, the combination is a transdermal patch accompanied by a faster-acting product such as gum, lozenges or an oral inhaler) is evaluated in the study by Brose et al., 2013 [18]. Nicotine is metabolized more rapidly in pregnancy, which theoretically suggests that the amount of NRT that is effective for smokers may not be effective for pregnant smokers. Therefore, it could be that a combination of various presentations of NRT is needed to confer a significant benefit in this second group, and its study is described here. At the four-week follow-up, 29.5% of participants (*n* = 1143) reported having been abstinent for at least two weeks and had an expired-air CO measurement of less than 10 ppm. In the trial, it describes that 29.4% of pregnant women who did not have medication continued to smoke, 31.6% of those who used only NRT and 21.2% of those who used combined NRT. Consistent with findings from previous RCTs in pregnancy, NRT alone is not associated with better abstinence rates; however, combined NRT was strongly associated with greater odds of abstinence at four-week follow-up (OR 1.93; 95% CI 1.13–3.29; *p* = 0.016), while NRT alone showed no benefit (OR 1.06; CI 95% from 0.60–1.86, *p* = 0.84). The trial by Brose et al., 2013 [18], is the only one in this review that provides evidence in favor of the efficacy of NRT during pregnancy.

### 3.3. Anomalies in Children of Mothers Who Consume NRT

As can be seen in the studies by Coleman et al., 2015 [34], Claire et al., 2020 [9], and Taylor et al., 2020 [22], an analysis of perinatal outcomes (preterm birth, low birth weight, and adverse reactions in the mother and baby at the time of delivery) is carried out, instead, only in the studies by Cooper et al., 2014 [32], Iyen et al., 2019 [33], Zhu et al., 2014 [35], and Dhalwani et al., 2015 [36], investigate how NRT could affect the offspring of mothers who consume this during pregnancy.

In Cooper et al. of 2014 [32], the offspring of women who use NRT during pregnancy seem to have a greater probability of presenting a development without problems at two years. However, five years later, the study by Iyen et al., 2019 [33] concludes that there is no evidence to support the hypothesis that the better infant development observed within infants born to women who were randomized to NRT in the trial was the result of quitting smoking induced by the use of nicotine patches.

The study by Zhu et al., 2014 [35] used data from the Danish National Birth Cohort to examine the association of ADHD with maternal and paternal smoking during pregnancy. If nicotine is responsible for this association, it can also be extrapolated to NRT consumers, so the association between the use of NRT by pregnant women during pregnancy, the spouse’s smoking and ADHD was investigated. In the investigation, of the 84,803 children, 2009 (2.4%) received a diagnosis of ADHD or medication for it during the follow-up period; it was also determined that the use of NRT by the mother during pregnancy is associated with a greater risk of ADHD in offspring than in those of mothers who did not smoke or use NRT.

The second study is that of Dhalwani et al., 2015 [36]; it does not study the effects of NRT on ADHD but rather evaluates the relationship between early pregnancy exposure to NRT or smoking with major congenital anomalies in the offspring. This latter study found that women exposed to NRT during early pregnancy had no increased risk of major congenital anomalies overall in their children compared with both women who did not smoke during pregnancy and women that did smoke. There were no statistically significant associations between maternal NRT use and system-specific anomalies except for respiratory anomalies.

### 3.4. NRT Review Articles

In the reviews found with these characteristics, no differentiation is made between faster-acting or long-acting NRT, so when the concept is used in these articles, reference is made to all possible presentations of NRT that have been used in published studies without distinctions. The first two studies, Coleman et al., 2015 [34], and Claire et al., 2020 [9], show that NRT used to quit smoking during pregnancy can increase abstinence rates. However, this evidence is of low certainty, as the effect was not apparent when potentially biased and uncontrolled RCTs in the placebo group were excluded from the analysis. In the subgroup analysis (placebo-controlled and non-placebo-controlled RCTs), performed in both reviews, there is no clear evidence in placebo-controlled RCTs (RR 1.21, 95% CI 0.95–1.55; six studies, 2063 women), while non-placebo-controlled studies did show evidence of a benefit (RR 8.55, 95% CI 2.05–35.71; three studies, 273 women).

In the trial by Claire et al., 2020 [9], a further subgroup analysis in which studies were grouped by the type of NRT used found no difference in the efficacy of NRT in those using patches or faster-acting NRT (test for subgroup differences *p* = 0.08).

In all three studies, Coleman et al., 2015 [34], Claire et al., 2020 [9], and Taylor et al., 2020 [22], found no evidence that NRT has a positive or negative impact on the health status of the newborn. There was no evidence of a difference between NRT and control groups in rates of miscarriage, stillbirth, preterm birth, birth weight, low birth weight, neonatal intensive care admissions, cesarean delivery, MCA or neonatal death. In all three studies, infants born to women who had been randomized to NRT had higher rates of “survival without developmental impairment” at two years compared with the placebo group [9,22,34].

Coleman et al. 2015 [34] and Claire et al. 2020 [9] show that NRT used to quit smoking during pregnancy can increase abstinence rates in this population group. However, this evidence is of low certainty as the effect was not apparent when potentially biased and non-placebo-controlled RCTs were excluded from the analysis. In the subgroup analysis (placebo-controlled and non-placebo-controlled RCTs), performed in both reviews, there is no clear evidence in placebo-controlled RCTs (RR 1.21, 95% CI 0.95–1.55; six studies, 2063 women), while non-placebo-controlled studies did show evidence of a benefit (RR 8.55, 95% CI 2.05–35.71; three studies, 273 women).

Table 1 shows the most important results and the methodological characteristics of the different selected articles. Some of these methodological characteristics include sample size and gestational period studied. The smoking and/or NRT-related parameters measured included: smoking habit, dependence, individualization, abstinence and security. The methodology and outcomes studied in each trial present a high degree of heterogeneity. There is currently no established standardized protocol to study the safety and/or efficacy of NRT in pregnancy.

## 4. Discussion

NRT is available as transdermal patches, lozenges, gum, sprays and inhalers. Patches provide long-acting nicotine exposure, while gum, lozenges, inhalers and sprays act faster with a shorter duration of action. Treatment may involve the use of a single formulation or multiple formulations simultaneously, usually a patch and a faster-acting product [37]. Nicotine gum is available on the market and makes it possible to obtain average levels of nicotine that exceed 5 ng/mL, the minimum figure necessary to achieve stimulation of nicotinic receptors and thereby reduce withdrawal symptoms in smokers [37,38]. Nicotine tablets are the form of NRT recommended for less dependent smokers [37,38]. Meanwhile, a nasal spray is ideal for heavy smokers who are highly dependent [37]. The nicotine inhaler is the Food and Drugs Administration (FDA)-approved formulation of NRT that most closely resembles smoking a cigarette, leading some smokers to find it more useful than other formulations [37].

Among the different presentations of NRT, whether they are faster-acting or long-acting, there is no significant evidence in favor of NRT in achieving abstinence among pregnant women [28,30,31,32]. Despite the low rates of abstinence obtained, in some of the studies, there is evidence of a decrease in cigarettes smoked from the start of the trial until the moment of delivery in favor of NRT [28,30]; in the rest of the studies [30,31], this parameter is not measured.

### 4.1. Safety

The results of the apparent safety of the use of NRT during pregnancy, as determined in the trials and reviews studied (except the trial by Dhalwani et al., 2015 [36]), contradict the classification that the FDA makes of the different NRT formulations during pregnancy; chewing gum and nicotine lozenges have been classified as category C in pregnancy (drugs for which a teratogenic risk cannot be ruled out, and their use should be restricted to those situations where there is no other safer drug). Other formulations (transdermal patches, inhalers and aerosol nicotine products) are classified as pregnancy category D (drugs that can cause teratogenicity), and the results of animal studies have shown that exposure to nicotine during pregnancy causes adverse effects on the endocrine, reproductive, respiratory, cardiovascular and neurological systems in the offspring. Regarding the safety results of the exposed studies, it is observed that in those who study short-acting NRT, there is a lower risk of premature delivery and higher birth weight among babies of pregnant women using NRT and not a placebo, which suggests that these types of formulations could be safe for the fetus [28].

After analyzing the results, we observe that NRT is not completely harmless for the fetus or for the mother; however, if an adequate assessment of the risk-benefit binomial is made, it does seem appropriate to use it to quit smoking during pregnancy, but it is necessary to establish recommendations on its use during this period.

### 4.2. Biological Plausibility

While it would be preferable for pregnant women to stop smoking without the need for NRT, if it is needed, it does not expose the fetus to a new drug that it would not already be exposed to otherwise and its use in place of cigarettes likely decreases the exposure to the other toxins and carbon monoxide present in cigarettes. This reduced exposure to carbon monoxide in the fetus could be the mechanism for some of the improved outcomes observed, such as birth weight, in NRT users.

Pregnancy is well known to affect the metabolism of some drugs and may result in higher or lower clearances compared to the non-pregnant state. If the metabolism of nicotine were slower during pregnancy, with the usual doses of NRT for adults, the plasma levels of nicotine could increase to toxic levels in the pregnant woman and potentially be more toxic to the fetus; however, the scientific literature affirms that in pregnancy the metabolism of this organic compound is accelerated [3,9,21,39,40]. Three clinical trials on nicotine metabolism during pregnancy synthesis are summarized in Table 2 [17,41,42].

The exact causes of increased metabolism during pregnancy are unknown, but more recent studies suggest the hypothesis that it may be due to increased progesterone levels during pregnancy. Non-pregnant women using progesterone-containing contraceptives metabolize nicotine more rapidly than women not using contraceptives; during pregnancy, these hormones begin to increase, so it is hypothesized that they may be responsible for the increased activity of CYP2A6 during pregnancy [15,43].

The hepatic enzyme cytochrome P450 2A6 (CYP2A6) is primarily responsible for converting nicotine to cotinine and cotinine to its close metabolite trans-3’-hydroxycotinine (3HC) [15,41,44]. The ratio of 3HC to cotinine (nicotine metabolite ratio (NMR)) is proportional to CYP2A6 activity, which is strongly correlated with total nicotine clearance, i.e., the higher the amount of 3HC, the lower the amount of cotinine, which translates into the increased metabolic activity of CYP2A6 [41].

↑CYP2A6 activity→ ↑NMR = (↑(3HC))/(↓(cotinine))

### 4.3. Limitations

Regarding the weaknesses of the study, we found that all the included studies were carried out in countries with a high economic level. Therefore, these results may not be applicable to lower-middle-income countries if women’s smoking patterns or beliefs about medication use in pregnancy differ.

In the reviewed studies, there is heterogeneity in the approach; hence, the lack of certainty regarding the efficacy of the therapy may have been the result of certain limitations in the way in which the studies presented have been developed. For example, while in some studies an individualization of the therapy doses is carried out based on the smoking status of each of the patients, in others, it is not, which could explain the lack of efficacy and low compliance by the participants. Moreover, during pregnancy, many women tend to reduce the number of cigarettes smoked per day [9], which leads us to think that possibly individualizing the dose of nicotine based on cigarettes smoked per day during pregnancy is not the most appropriate method to achieve a 100% substitution of the nicotinic needs of pregnant women. Knowing the patient’s smoking status is the first measure that should be taken by health personnel prior to the start of any intervention [10]. In the studies presented, the increased metabolism of nicotine during pregnancy, which will cause pregnant women to require higher levels of nicotine to satisfy their needs, is not considered and could be a factor in the lack of success in smoking cessation in the studies. Cotinine is going to be a reliable biological marker, but during pregnancy, the metabolism of nicotine is increased, so the levels of cotinine in saliva are also going to be decreased; therefore, adjusting the dose based on salivary cotinine levels does not seem to be the most effective method to obtain 100% nicotine substitution in pregnant women either.

## 5. Conclusions

This review provides an overview of studies on the use of NRT during pregnancy. Despite the fact that there is an increase in studies that aim to evaluate the use of NRT during pregnancy, the number of studies continues to be very low. Although the studies have certain weaknesses, the information they provide on the subject is essential to be able to establish guidelines and protocols for the management of smoking cessation during pregnancy. The combination of faster-acting and long-acting NRT has been the only strategy that has achieved higher rates of abstinence among pregnant women. Faster-acting formulations seem to be the safest and even most beneficial forms for the offspring. Further, well-designed controlled clinical trials evaluating safety based on other parameters that are not exclusively perinatal results are suggested. The use of NRT during pregnancy must involve the active participation of health personnel to guarantee its correct use as an individualization of the formulation. Moreover, doses must be carried out for each patient based on their individual nicotinic needs, taking into account both psychosocial factors and the increased metabolism of nicotine during pregnancy before starting treatment.

## Figures and Tables

**Table 1:** Summary of selected articles on the use of nicotine replacement therapy during pregnancy.

	Characteristics		Smoking and/or NRT-Related Parameters Measured													Results	
	Sample Size	GestationalWeek	Smoking Habit				Dependence		Individualization			Abstinence		Security		Efficacy	Safety
			CPD		CO	COT	FTND	HSI	Yes		No	CO	COT	Perinatal	2-Years		
			Before	During					CPD	COT							
Oncken et al., 2008 [28]	194	17	17	10	✕	✕	✕	-	✕	-	-	✕	✕	✕	-	✕	✓
Oncken et al., 2019 [29]	137	17–19	?	8	✕	✕	✕	-	✕	-	-	✕	✕	✕	-	✕	✓
Coleman et al., 2012 [30]	1050	16	20	14	-	✕	-	-	-	-	✕	✕	✕	✕	-	✕	≈
Berlin et al., 2014 [31]	402	12–20	10	5	✕	✕	✕	-	-	✕		✕	✕	✕	-	✕	≈
Cooper et al., 2014 [32]	891	-	-	-	-	-	-	-	-	-	-	-	-	-	✕	-	✓
Iyen et al., 2019 [33]	884	-	-	-	-	-	-	-	-	-	-	-	-	-	✕	-	✕
Brose et al., 2013 [18]	3880	>12	?	?	✕	-	-	✕	?	?	-	✕	-	-	-	✓	-
Zhu et al., 2014 [35]	84803	-	-	-	-	-	-	-	-	-	-	-	-	-	✕	-	✕
Dhalwani et al., 2015 [36]	192498	-	-	-	-	-	-	-	-	-	-	-	-	✕	-	-	✓
Coleman et al., 2015 [34]	2210	-	-	-	-	-	-	-	-	-	-	-	-	✕	✕	✓/✕	✓
Claire et al., 2020 [9]	2412	-	-	-	-	-	-	-	-	-	-	-	-	✕	✕	✓/✕	✓
Taylor et al., 2020 [22]	-	-	-	-	-	-	-	-	-	-	-	-	-	✕	✕	-	✓

CPD: Cigarettes per day. CO: Carbon monoxide. COT: Cotinine. FTND: Fagerström test for nicotine dependence. HSI: Heavy smoking index. ✕: Not correctly evaluated (heterogeny in design approach and/or no clearly defined criteria or standards and/or no individualized NRT dose and/or no consideration of metabolic nicotine response and/or smoking habit/reduction evaluated solely by number of cigarettes smoked per day). ✓: Correctly evaluated. ≈: Partially correctly evaluated. ?: Information provided in the article is unclear. -: Information is not available in the article.

**Table 2:** Summary of the clinical trials on nicotine metabolism during pregnancy.

	Sample Size	Information Used	Main Outcomes
Dempsey et al., 2002 [42]	Blood and urine (10 pregnant women)	Cl (renal/non-renal): nicotine and cotinine	↑ Cl (nicotine and cotinine) ↑ t1/2 cotinine (8.8 vs. 16.6)
Arger et al., 2019 [41]	Urine (47 pregnant women in a smoking cessation RCT)	Fast metabolism: Urinary NMR ([3HC]/[cotinine])	↑ NMR during early and late pregnancy compared to postpartum
Gwon et al., 2021 [17]	Saliva (43 pregnant women):—light smokers—heavy smokers	Cotinine levels in (1st trimester (T1)—3rd trimester (T2)—postpartum (T3))	Differences in cotinine levels in maternal saliva at different time points and between the two groups of smokers.

RCT: Randomized Clinical Trial. Cl: Clearance. NMR: Nicotine Metabolism Ratio. 3HC: trans-3′-hidroxicotinina. T: Trimester of Pregnancy.

## Data Availability

Not applicable.
